# Bioactive Films Based on Starch from White, Red, and Black Rice to Food Application

**DOI:** 10.3390/polym14040835

**Published:** 2022-02-21

**Authors:** Luan Ramos da Silva, José Ignacio Velasco, Farayde Matta Fakhouri

**Affiliations:** 1 Faculty of Engineering, Federal University of Grande Dourados (FAEN/UFGD), Dourados 79804-970, Brazil; luanramosea@gmail.com; 2 Faculty of Food Engineering, University of Campinas (FEA/UNICAMP), Campinas 13083-970, Brazil; 3 Poly2 Group, Department of Materials Science and Engineering, Universitat Politècnica de Catalunya (UPC BarcelonaTech), Calle Colon, 11, 08221 Terrassa, Spain; jose.ignacio.velasco@upc.edu

**Keywords:** *Oryza sativa*, biodegradable packages, polymer from renewable source

## Abstract

Packages from renewable sources have been the focus of many studies, due to the consumer needs for high-quality food, environmental concern related to the inadequate discard of packaging, low percentage of packaging recycling, and starch application by a viable method. Thus, this work aimed to develop bioactive packages based on white, red, and black rice starch and analyze the influence of macromolecule and plasticizer type, even its blends, on the characteristics of films. Films were characterized by color, opacity, thickness, water solubility, water vapor permeability, and bioactive properties. The use of rice starch in the development of edible and/or biodegradable films was feasible, with all the formulations tested presenting a homogeneous matrix and the films obtained varying in hue, to the naked eye, as a function of the starch used. Variation of the type of starch and plasticizer, as well as the concentrations of the same, resulted in films with differences in all studied properties. Films prepared with 5% of starch and 30% of sorbitol showed phenolic compounds and antioxidant capacity, using the DPPH and ABTS methods, indicating that these can be considered bioactive packages and also suitable for food application.

## 1. Introduction

Rice (*Oryza sativa*) is considered a source of income and nutrients to many people around the world, especially in Asian countries [1]. The success of this cereal is associated with its nutritional value and quality, its practicality and applicability, and its characteristics of hypoallergenic and nontoxic to celiac, which makes it a great substitute for wheat, to develop gluten-free products [2].

The most consumed kind of rice is the polished white type; however, there are around 120,000 varieties of rice known worldwide [3]. Therefore, pigmented varieties of rice are gaining attention from researchers, due to its beneficial properties for human health. They may contribute to the reduction of chronic disease development, such as cardiovascular, Type 2 diabetes, obesity, and cancer [4]. Those properties are results of the grain composition, which is rich in health-promoting compounds such as vitamins, minerals, phytochemicals, and phenolic compounds [5,6,7,8,9].

The high quantity of starch in rice grains (±80%) makes it suitable for industrial use. A large amount of starch can be found on rice residues from its processing, such as broken rice, which may be considered as raw material for starch extraction. The use of a wasted material goes towards the global requirements for reducing the waste of natural resources [10].

Rice starch is known by its small granules, which can be used on cosmetic applications, and in textile, food, or materials industry [9]. The industrial use of starch, in the development of materials, is increasing due to research that show it as a promising material to produce food packaging. This material can change its characteristics by many factors, such as the source. Thereby, researchers are studying starches from alternative sources to indicate an appropriate application for them [11,12,13,14,15].

There are many materials that can be used in the development of packaging, such as whey protein isolate [16], soy protein isolate [17], or starch [11]. In addition, the raw material for this kind of packaging could be obtained from many sources, such as bacteria [18], cow milk [19], biomass [10], and others. Thereby, starch is a polymer suitable to be applied in many kinds of food packaging, such as rigid [20], foam [21], flexible by casting [22], or by thermoplastic extrusion followed by blowing [23]. Specifically in flexible packaging, there are edible and biodegradable films, which are considered sustainable packaging systems. This is due to the lack of waste during the process, its biodegradability, and its promotion to increase the shelf life of food products that contribute to reduce food waste.

Edible and/or biodegradable films have to be thin, and they are used to pack many food products which can be eaten together. They are made from a filmogenic solution, formed by a macromolecule, a solvent (usually water), a plasticizer agent, and, if necessary, pH adjusters or additives (capable of adding functional properties to the packaging) [24]. Other materials can be added into the formulation to improve its properties, such as cellulose nanofibers [25].

The edible films could confer many functional advantages to their products, mainly when they have some antimicrobial or antioxidant compounds in the composition [26,27,28]. Studies of those packaging products have been explored by researchers due to the need of consumers for high-quality products, the increase in the concern about environmental impacts, inappropriate disposal, low recycling tax of packaging, and the application of starch in viable process.

Flores et al. [29] analyzed the influence of film-forming methods and the use of potassium sorbate on the physical properties of starch edible films, and they observed that changes in the process modify the final properties of packaging. In addition, potassium sorbate added in the formulation resulted in changes in physical characteristics, except for solubility. Thus, we can see the importance of controlling the process of edible film development, considering simple modifications could cause reduction in the barrier, mechanical, or thermal properties.

Those films that can offer any bioactivity for the packaged food or in its consumption, are considered as bioactive films. Therefore, many materials, natural or not, could be inserted in its formulation to give those special properties. Moreover, researchers are obtaining them from food waste, essential oils, extracts, and others [30,31,32,33,34]. However, the use of a matrix, which already is a source of bioactive compounds, may work in this process.

Priyadarshi et al. [35] developed edible coating incorporated with grape seed extract, and the extract contributed with antioxidant and antimicrobial compounds, which made it possible to prevent lipid oxidation and extend the shelf life of peanuts. Furthermore, Maniglia et al. [36] developed films with babassu starch and had no need of added bioactive compounds, due to the rich composition of the used starch, and the obtained films presented a great antioxidant capacity. In addition, Wang et al. [37] developed a coating based on protein nanofibers and polyphenols and applied it to duck egg yolks. This demonstrates the versatility of those packaging products, which could be used for many products, and could be made from different materials.

The COVID pandemic has changed our behavior in society, and packaging that presents some bioactivity may be an alternative solution for transportation of quality food [38]. Therefore, the aims of this work were to develop bioactive packaging based on starch of white, red, and black rice, and analyze the influence of starch and plasticizer type and concentration on the film characteristics.

## 2. Materials and Methods

### 2.1. Materials

White, red, and black rice (Armazém Santa Filomena, São Paulo, Brazil) were used as a source of starch. Sorbitol D (Dinâmica Química Contemporânea LTDA, São Paulo, Brazil) and glycerol P.A. (Synth, São Paulo, Brazil) were used as plasticizer. Calcium chloride PA (Sigma-Aldrich, Saint Louiz, MI, USA), gallic acid (Dinâmica Química Contemporânea LTDA, São Paulo, Brazil), acetone PA (Dinâmica Química Contemporânea LTDA, São Paulo, Brazil), methanol PA (Dinâmica Química Contemporânea LTDA, São Paulo, Brazil), ethanol PA (Dinâmica Química Contemporânea LTDA, São Paulo, Brazil), DPPH (1,1-difenil-2-picril-hidrazil) (Sigma-Aldrich, Saint Louiz, MI, USA), ABTS [2,2-azinobis (3-ethylbenzothiazoline-6-sulfonic acid)] (Sigma-Aldrich, Saint Louiz, MI, USA), and Folin–Ciocalteau reagent (Dinâmica Química Contemporânea LTDA, São Paulo, Brazil) were used for analyses.

### 2.2. Methods

#### 2.2.1. Starch Extraction

Starches were extracted from white, red, and black rice, by wet extraction method. The varieties of rice were submitted to wet extraction in order to obtain the starches. Firstly, the raw material was washed and weighed. The rice was submitted to disintegration in water (1:2 rice: water) in an industrial blender (Model SPL-049, SPOLU, Itajobi, SP, Brazil) for 5 min. The disintegrated material was filtered in order to separate the residues, which were again disintegrated and filtered. The filtered liquid was purified with a strainer (mesh 100/0.149 mm) and placed on a bench for 24 h to decant the starch. The starch was dried in an oven with air circulation (NG Científica, Campo Grande, Brazil) at 45 °C for 10 h, and stored in plastic bags, in the dark. To extract starch from red rice, the filter needed to be centrifuged at 1100 rpm (G force = 318× *g*) for 15 min using a Simplex II centrifuge (ITR, Esteio, RS, Brazil). Starch from white, red, and black rice presented an amylose content of 18.61, 25.75, and 20.02%, respectively, and pasting temperature ranged from 79.0 to 89.0 °C [9].

#### 2.2.2. Film Production

Rice starch films were carried out by casting technique. Twelve formulations of film (for each starch) were developed, varying starch quantity and plasticizer type and quantity (Table 1), resulting in 36 formulations. For each formulation, the starch and plasticizer were weighed in an analytical balance (PA214P, Ohaus, Nänikon, Switzerland) and distilled water added. The mixture was heated to 85 °C at a thermostatic bath (550, Fisatom, São Paulo, Brazil) for 5 min. Aliquots of 30 mL of filmogenic solution were placed in Petri plates (15 cm diameter). Drying process was carried out at 18 °C for 30 h. The obtained films were placed in glass desiccator, at 25 °C and 56% of relative humidity until the analyses.

#### 2.2.3. Color and Opacity

Instrumental color of films was carried out by direct reading by digital colorimeter CR 400 (Konica Minolta), on white and black backgrounds. The following were measured: L* value (luminosity parameter) ranging from black (L* = 0) to white (L* = 100); a* value, which range from green (−a*) to red (+a*); and b* value, which range from blue (−b*) to yellow (+b*). Chromaticity (C*) is the relation between a* and b* values, resulting in the real color of the analyzed object (Equation (1)). The readings were realized in five different points in both backgrounds. Using L* values on white (L*_w_) and black (L*_b_) backgrounds, the opacity of films was calculated (Equation (2)).

(1) $$C*=\sqrt{a^{2}+b^{2}}$$

(2) $$Opacity\;\left(\%\right)=\frac{L_{b}^{*}}{L_{w}^{*}}\times 100$$

#### 2.2.4. Thickness

The film thickness was carried out by a micrometer (MDC 25 M, Mitutoyo, Sakado, Japan), ± 0.001 mm of accuracy, in fifteen different points of each sample.

#### 2.2.5. Water Solubility

Water solubility of films was carried out according to Gontard, Guilbert, and Cuq [39]. Film samples were cut into disks of 2 cm in diameter, in triplicate, dried at 105 °C for 24 h, in an oven with air circulation (MA035/1, Marconi, São Paulo, Brazil), and weighed. The dehydrated samples were individually immersed in 50 mL of distilled water, and maintained under mechanical agitation (75 rpm) for 24 h at 25 °C. After this period, not-solubilized samples were removed and dried (105 °C for 24 h). Solubility was expressed according to Equation (3).

(3) $$Solubility\;\left(\%\right)=\frac{M_{i}-M_{f}}{M_{i}}\times 100$$

in which *M_i_* is the initial dry mass of the films (g), and *M_f_* is the final dry mass of nonsolubilized films (g).

#### 2.2.6. Water Vapor Permeability

Water vapor permeability rate of the films was determined gravimetrically based on ASTM E96 method [40], using an acrylic cell, with a central opening (diameter of 4.3 cm), in which the film was fixed. The bottom of the cell was filled with 10 g of calcium chloride PA, creating a dry environment inside (0% relative humidity at 25 °C). This cell was placed in a desiccator containing saturated sodium chloride (75 ± 3% RH at 25 °C).

Water vapor transferred through the film was determined by mass gain of calcium chloride in 24 h. The film thickness consisted of the average of five random measurements made on different parts of the film. The effect of air, as described by McHugh and Krochta [41] and Gennadios, Weller, and Testin [42], between the bottom of the film and calcium chloride surface was not considered when calculating the water vapor transfer rate. Tests were performed in triplicate. Samples were placed on a desiccator at 56% RH/25 °C, 48 h before the test.

(4) $$WVP=\frac{e}{A\times \Delta p}\times M$$

in which *WVP* is water vapor permeability (g mm/m^2^ dkPa), *e* is mean film thickness (mm), *A* is permeation area (m^2^), ∆*p* is partial vapor pressure difference between two sides of film (kPa, at 25 °C), and *M* is absorbed moisture rate, calculated by linear regression of weight gain and time, in steady state (g/day).

#### 2.2.7. Bioactive Compounds and Antioxidant Activity

For phenolic compounds and antioxidant activity, film extracts (W10, R10, and B10) were prepared according to Rufino et al. [43]. An aliquot of 3 g of each sample was submitted to extraction with 40 mL of methanol 50%, and they were homogenized and kept resting for 1 h at room temperature (25 °C). Then, they were submitted to centrifugation at 1100 rpm (G force = 318× *g*) for 30 min using a Simplex II centrifuge (ITR, Esteio, RS, Brazil). The supernatants were placed in volumetric flasks of 100 mL, and the residues were submitted to another extraction with 40 mL of acetone 70%, homogenized, and kept resting for 1 h at room temperature (25 °C). They were centrifuged at 1100 rpm (G force = 318× *g*) for 30 min using a Simplex II centrifuge (ITR, Esteio, RS, Brazil), the supernatants were transferred to the volumetric flask, and the volume was completed to 100 mL with distillated water.

The determination of total phenolic compounds follows the Folin and Ciocalteu [44] method, as developed by Singleton and Rossi [45], in triplicate. Gallic acid was used as a standard, and results were expressed in mg GAE/100 g.

Antioxidant activity was evaluated by DPPH method [43], in a spectrophotometer (model 7310, Jenway, Staffordshire, UK) at 515 nm, expressed in g/g of DPPH, and by ABTS method [46], in a spectrophotometer (model 7310, Jenway, Staffordshire, UK) at 734 nm, expressed by µM trolox/g. Both analyses were performed in triplicate.

#### 2.2.8. Statistical Analysis

Data were submitted to analysis of variance (ANOVA) and compared by the Tukey test, at 5% significance level, with STATISTICA 8.0 software (Statsoft, Tulsa, OK, USA).

## 3. Results and Discussion

### 3.1. Visual Aspects and Physical Analysis

**Figure 1:**
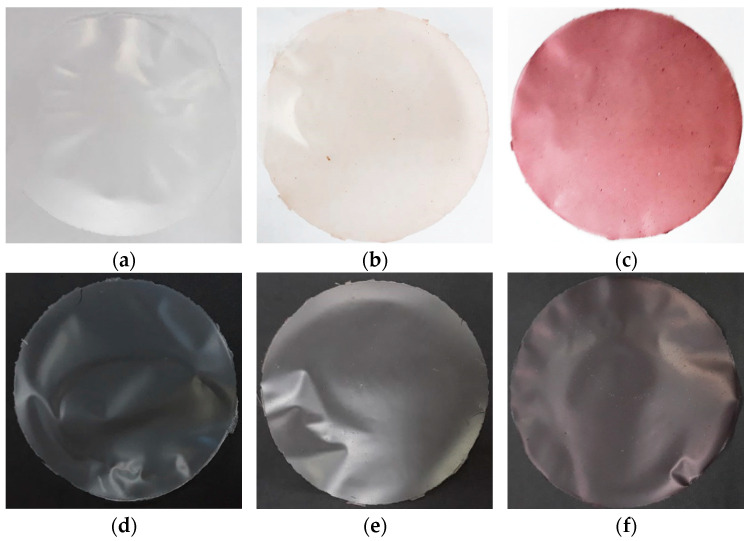
Elaborated films with 5% of starch + 30% of sorbitol. Film based on white (**a**,**d**), red (**b**,**e**), and black (**c**,**f**) rice starch.

The developed films demonstrated great film formation, homogeneity, and no rupture, fissure zone, or insoluble particles (Figure 1). Films of red rice starch were easier to remove from the plates. This may be justified by the amylose content, which plays an important role in film formation [11,22], resulting in stronger and more flexible films when higher [47]. The studied starches present amylose content of 25.75%, 20.02%, and 18.61% for red, black, and white rice [9]. Films of white, red, and black rice starch presented whitish, pinkish, and purple coloration, respectively, to the naked eye, and these colors represent the starch color (Figure 1).

The color difference between films based on rice starch can be seen in Table 2. Higher values of luminosity were observed for films of white rice starch (92.61–94.16), while those made of red rice starch presented luminosity between 82.63 and 87.82. The lowest luminosity values were from films of black rice starch, ranging from 53.10 to 66.92. Therefore, among the starches, the black one presented a higher variation of L*. Starch concentration on films does not affect the luminosity of them. Otherwise, plasticizer significantly influenced formulations with 3% of starch and 25% of plasticizer, and sorbitol contributed to increase this parameter on the packaging. In general, L* value decreased in higher quantities of starch for all studied plasticizers. For black rice starch films, sorbitol produced packaging with higher L* values, unlike Maniglia et al. [48] in babassu starch films with different plasticizers, which showed lower or same L* values to those made with sorbitol, compared to glycerol. This demonstrates that the plasticizers have different behaviors in distinct starches.

The increase in the starch quantity promoted positive changes of a* chroma in all film formulations. This indicates, in films based on red rice starch, that higher concentrations of starch can result in more reddish packaging, as can be seen by the naked eye (Figure 1). Homez-Jara et al. [49] verified an increase in a* chroma with the polymer increase for chitosan-based films. The same influence was not observed for b* chroma, which presented its lowest value (4.23) for the packaging with 3 g of starch and 25% glycerol (W2), while the highest value was seen for the sample with 5 g of starch and 25% sorbitol + glycerol (R9 = 11.15). Among the studied films, those made by red rice starch presented higher values of b* chroma, followed by films based on black and white rice starch.

The highest values of chromaticity were observed in films based on black rice starch, ranging from 12.74 (B1) to 18.82 (B10). A low variation occurred for this parameter when related to starch mass, type, and content of plasticizer. However, a high color saturation was seen on the samples with higher starch content.

Films made of black rice starch demonstrated higher values of opacity when compared to those elaborated with white and red rice starch. In films made of white rice starch, the starch mass significantly influenced the opacity of them. Maniglia et al. [48], studying babassu starch films with different plasticizers, obtained lower values of opacity, when compared to our results, ranging from 2.9 to 7.0%. This means that our packaging varieties are more suitable to be applied in photosensitive products.

The starch mass difference significantly influenced the thickness of all films formulations, where those made with higher concentrations of starch presented higher thickness (Table 2). Laohakunjit and Noomhorm [50] obtained films based on rice starch with thickness between 0.105 and 0.113 mm, which are higher than our results, and it is justified by the quantity of material used for film formation, since the amount of solids significantly influences this parameter in flexible films [51]. In addition, the thickness of films may influence the application of them [52,53]. Plasticizer type did not influence the thickness of the studied packaging.

### 3.2. Water Solubility and Water Vapor Permeability of Films

The films elaborated with white, red, and black rice starch presented higher water solubility in formulations with sorbitol as plasticizer, and lower in those with glycerol (Table 3). Formulations plasticized with the blend demonstrated intermediate results for water solubility.

Changes in macromolecule and plasticizer concentration on film formulations and, consequently, their thickness significantly influenced the water solubility of our films. Higher values were observed with the increase in concentrations for all starch studied. The highest water solubilities were observed on formulations W10 (20.09%), R10 (20.66%), and B7 (28.14%). These results are important for determining the film applications. Maniglia et al. [36] observed lower water solubility for films made with babassu starch extracted with water and glycerol (24.3%), when compared with sorbitol (34.4%).

Our results corroborate other studies [11,54], and films with sorbitol as plasticizer have shown lower values of water vapor permeability, when compared with those elaborated with glycerol, for all studied starches (Table 3). Rodrígues et al. [55], studying starch films with or without glycerol, verified a significant increase in water vapor permeability when the plasticizer was added.

White rice starch films presented water vapor permeability between 0.32 and 7.35 g mm/m^2^ dkPa (Table 3), whereas those based on red or black rice starch ranged from 0.94 to 12.83 g mm/m^2^ dkPa and 0.60 to 15.75 g mm/m^2^ dkPa, respectively. Films produced by different sources tend to present different characteristics [12,56]. Flexible films produced with 5% of maize, cassava, and wheat starch and 30% of glycerol presented water vapor permeability of 9.60, 5.04, and 10.80 g mm/m^2^ dkPa, respectively [12].

### 3.3. Bioactive Compounds and Antioxidant Activity

For bioactive compounds and antioxidant activity, we selected films with the highest water solubility and lowest water vapor permeability; therefore, formulations with 5% of starch and 30% of sorbitol were chosen. Films based on studied rice starches are significantly different in phenolic compounds content, being 0.58 ± 0.02, 25.26 ± 0.87 and 164.96 ± 0.25 mg EAG/100 g, for films of white, red, and black rice starch, respectively (Table 4). The difference is associated with phenolic compounds content in the starches, which presented the same tendency, presenting, from aqueous extract, 288.95, 478.46, and 661.60 mg EAG/100 g of starch from white, red, and black rice, respectively [9].

**Figure 2:**
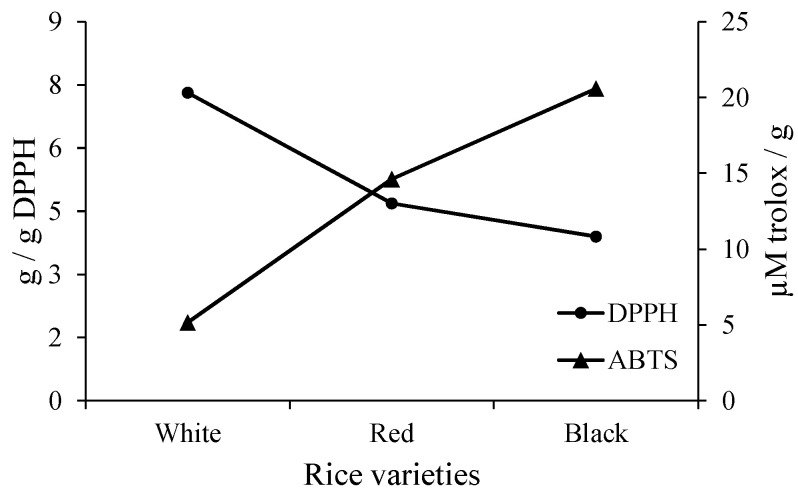
Antioxidant activity (DPPH and ABTS) of films based on white, red, and black rice starch (5% starch + 30% sorbitol).

Antioxidant capacity of films, by DPPH and ABTS, are shown at Figure 2. The obtained results, from both methods, corroborate among them. Due to DPPH, lower values mean high antioxidant activity, whereas the opposite explain ABTS results.

The starches were submitted to many processes, from the extraction to the film obtaining, and still resulted in packaging with antioxidant activity. Thus, black rice starch films presented the highest antioxidant capacity when compared to others. Many works have developed bioactive films based on the incorporation of some additives, such as extracts [28], oils [14], or pulp [11].

Bioactive packaging with antioxidant activities can be applied in many products, protecting them against free radicals. In addition, as an edible packaging, it can contribute to consumer health. Nogueira et al. [51], studying films based on arrowroot starch, demonstrated that when using blackberry microcapsules on filmogenic solution, the packaging become bioactive. Nevertheless, our films do not need any additive to present antioxidant capacity. The same was seen on babassu films with different plasticizers [48].

The developed bioactive films present some advantages, such as the elaboration of them without the need for incorporation of any other source of bioactive compounds, which contributes to reduction in production costs; the use of starch from an abundant raw material that could be obtained from its byproducts, which can contribute to the environmental issue by reducing waste; and the application of this packaging in food products, which could extend its shelf life and reduce food loss.

## 4. Conclusions

The use of starch from studied varieties of rice in the development of edible and/or biodegradable films is viable, as all tested formulations presented film formation, homogeneity, and no ruptures or insoluble particles.

Changes in type of starch and plasticizer, as well as their concentrations, resulted in films with differences in opacity, thickness, water solubility, and water vapor permeability.

Films made of 5% of starch and 30% of sorbitol presented phenolic compounds and antioxidant capacity using DPPH and ABTS methods; thus, they can be considered bioactive packaging, and could be used by the food industry. However, the interaction of bioactive compounds and food matrix should be tested to prove the efficiency of them.

## Figures and Tables

**Table 1:** Formulations of films to each variety of rice: white (W), red (R), and black (B).

Code			Starch Quantity (g) *	Plasticizer Type	Plasticizer Quantity (%) **
White	Red	Black			
W1	R1	B1	3	Sorbitol	25
W2	R2	B2	3	Glycerol	25
W3	R3	B3	3	Sorbitol + Glycerol (1:1)	25
W4	R4	B4	3	Sorbitol	30
W5	R5	B5	3	Glycerol	30
W6	R6	B6	3	Sorbitol + Glycerol (1:1)	30
W7	R7	B7	5	Sorbitol	25
W8	R8	B8	5	Glycerol	25
W9	R9	B9	5	Sorbitol + Glycerol (1:1)	25
W10	R10	B10	5	Sorbitol	30
W11	R11	B11	5	Glycerol	30
W12	R12	B12	5	Sorbitol + Glycerol (1:1)	30

* Quantity of starch to 100 mL of distilled water. ** Quantity of plasticizer as a function of starch mass.

**Table 2:** Physical properties of films based on white, red, and black rice starch.

Code	L*	a*	b*	C*	Opacity (%)	Thickness (mm)
W1	94.16 ± 0.70 ^a^	−0.34 ± 0.02 ^ab^	4.45 ± 0.12 ^bc^	4.39 ± 0.12 ^bc^	45.42 ± 0.53 ^ac^	0.068 ± 0.006 ^b^
W2	93.20 ± 0.45 ^bc^	−0.37 ± 0.02 ^abc^	4.23 ± 0.04 ^e^	4.27 ± 0.04 ^d^	45.76 ± 0.70 ^a^	0.075 ± 0.005 ^b^
W3	92.52 ± 0.45 ^c^	−0.33 ± 0.03 ^a^	4.31 ± 0.06 ^ce^	4.32 ± 0.06 ^cd^	45.68 ± 0.40 ^ab^	0.078 ± 0.007 ^b^
W4	92.95 ± 0.51 ^b^	−0.38 ± 0.03 ^bcd^	4.29 ± 0.04 ^de^	4.31 ± 0.04 ^de^	45.95 ± 0.45 ^a^	0.075 ± 0.006 ^b^
W5	93.37 ± 0.35 ^ab^	−0.36 ± 0.02 ^abc^	4.83 ± 0.08 ^a^	4.85 ± 0.08 ^a^	44.87 ± 0.44 ^bc^	0.078 ± 0.005 ^b^
W6	94.15 ± 0.41 ^a^	−0.38 ± 0.01 ^bcd^	4.53 ± 0.06 ^b^	4.56 ± 0.06 ^b^	44.72 ± 0.16 ^c^	0.071 ± 0.007 ^b^
W7	92.64 ± 0.18 ^bc^	−0.40 ± 0.01 ^cde^	4.38 ± 0.09 ^cd^	4.43 ± 0.09 ^ce^	37.61 ± 0.31 ^d^	0.104 ± 0.008 ^a^
W8	93.03 ± 0.31 ^bc^	−0.43 ± 0.01 ^efg^	4.44 ± 0.04 ^bc^	4.45 ± 0.04 ^bc^	37.38 ± 0.37 ^d^	0.107 ± 0.006 ^a^
W9	92.61 ± 0.23 ^bc^	−0.43 ± 0.02 ^efg^	4.52 ± 0.05 ^b^	4.55 ± 0.05 ^b^	37.66 ± 0.18 ^d^	0.104 ± 0.005 ^a^
W10	93.05 ± 0.15 ^bc^	−0.42 ± 0.01 ^df^	4.44 ± 0.02 ^bc^	4.45 ± 0.02 ^bc^	37.46 ± 0.18 ^d^	0.101 ± 0.004 ^a^
W11	93.15 ± 0.21 ^bc^	−0.46 ± 0.02 ^efg^	4.56 ± 0.05 ^b^	4.58 ± 0.05 ^b^	37.94 ± 0.37 ^d^	0.095 ± 0.005 ^a^
W12	93.07 ± 0.24 ^bc^	−0.46 ± 0.01 ^g^	4.52 ± 0.02 ^b^	4.54 ± 0.02 ^b^	37.83 ± 0.10 ^d^	0.096 ± 0.003 ^a^
R1	87.10 ± 0.79 ^a^	2.91 ± 0.35 ^ce^	8.70 ± 0.40 ^de^	2.98 ± 0.34 ^cde^	39.06 ± 0.60 ^cde^	0.071 ± 0.005 ^e^
R2	86.45 ± 1.23 ^ab^	2.75 ± 0.34 ^ce^	8.94 ± 0.24 ^cd^	2.79 ± 0.33 ^df^	38.38 ± 0.65 ^de^	0.079 ± 0.009 ^ce^
R3	87.04 ± 0.87 ^a^	2.29 ± 0.19 ^e^	8.10 ± 0.27 ^e^	2.24 ± 0.19 ^ef^	39.18 ± 0.42 ^cd^	0.080 ± 0.009 ^be^
R4	87.82 ± 0.95 ^a^	2.13 ± 0.24 ^e^	7.97 ± 0.33 ^e^	2.09 ± 0.24 ^f^	37.92 ± 0.53 ^e^	0.070 ± 0.004 ^e^
R5	87.23 ± 0.84 ^a^	2.24 ± 0.36 ^e^	8.30 ± 0.45 ^de^	2.30 ± 0.37 ^ef^	36.75 ± 0.97 ^f^	0.073 ± 0.008 ^de^
R6	86.28 ± 0.42 ^ab^	2.57 ± 0.20 ^de^	8.54 ± 0.30 ^de^	2.68 ± 0.19 ^def^	38.84 ± 0.17 ^cde^	0.073 ± 0.009 ^de^
R7	84.92 ± 0.30 ^b^	3.72 ± 0.14 ^ab^	10.21 ± 0.23 ^b^	3.83 ± 0.13 ^ab^	40.49 ± 0.17 ^ab^	0.099 ± 0.007 ^abc^
R8	84.96 ± 0.64 ^b^	3.95 ± 0.57 ^ab^	10.23 ± 0.49 ^b^	3.73 ± 0.56 ^ab^	39.65 ± 0.37 ^bc^	0.093 ± 0.009 ^bcd^
R9	82.63 ± 0.27 ^c^	4.47 ± 0.21 ^a^	11.15 ± 0.30 ^a^	4.54 ± 0.20 ^a^	40.98 ± 0.18 ^a^	0.087 ± 0.005 ^be^
R10	85.30 ± 0.53 ^b^	3.34 ± 0.24 ^bcd^	9.65 ± 0.33 ^bc^	3.39 ± 0.24 ^bcd^	39.65 ± 0.30 ^bc^	0.101 ± 0.007 ^ab^
R11	85.29 ± 0.78 ^b^	3.42 ± 0.27 ^bc^	9.86 ± 0.34 ^b^	3.29 ± 0.27 ^bcd^	39.27 ± 0.90 ^cd^	0.101 ± 0.007 ^ab^
R12	86.37 ± 0.35 ^ab^	3.54 ± 0.76 ^bc^	9.73 ± 0.62 ^bc^	3.11 ± 0.75 ^bc^	38.86 ± 0.22 ^cde^	0.118 ± 0.005 ^a^
B1	66.92 ± 0.61 ^a^	11.83 ± 0.67 ^f^	5.34 ± 0.10 ^d^	12.74 ± 0.62 ^f^	44.40 ± 0.58 ^ef^	0.062 ± 0.008 ^e^
B2	60.97 ± 0.25 ^c^	13.13 ± 0.68 ^def^	6.45 ± 0.43 ^ab^	14.57 ± 0.46 ^de^	47.17 ± 0.64 ^d^	0.062 ± 0.006 ^e^
B3	59.40 ± 0.62 ^d^	13.99 ± 0.36 ^de^	6.44 ± 0.57 ^ab^	15.32 ± 0.23 ^cd^	46.87 ± 0.62 ^d^	0.071 ± 0.008 ^ce^
B4	65.87 ± 0.95 ^ab^	12.72 ± 0.58 ^ef^	5.64 ± 0.19 ^cd^	14.10 ± 0.56 ^ef^	45.09 ± 0.59 ^e^	0.069 ± 0.006 ^de^
B5	61.22 ± 0.89 ^e^	13.37 ± 0.69 ^de^	6.07 ± 0.31 ^bc^	14.71 ± 0.56 ^de^	46.89 ± 0.72 ^d^	0.076 ± 0.007 ^bce^
B6	57.28 ± 0.67 ^ef^	14.45 ± 0.30 ^cd^	5.64 ± 0.41 ^cd^	15.66 ± 0.69 ^cd^	44.34 ± 0.69 ^ef^	0.070 ± 0.008 ^ce^
B7	65.33 ± 0.39 ^b^	15.68 ± 0.89 ^bc^	5.65 ± 0.19 ^cd^	16.43 ± 0.53 ^bc^	43.32 ± 0.53 ^f^	0.086 ± 0.009 ^acd^
B8	57.76 ± 0.56 ^e^	16.15 ± 0.14 ^ab^	6.64 ± 0.02 ^ab^	17.49 ± 0.52 ^ab^	49.02 ± 0.52 ^c^	0.085 ± 0.004 ^acd^
B9	56.03 ± 0.50 ^fg^	16.88 ± 0.66 ^ab^	6.54 ± 0.11 ^ab^	17.98 ± 0.70 ^a^	51.21 ± 0.70 ^b^	0.090 ± 0.009 ^ac^
B10	53.10 ± 0.59 ^h^	17.42 ± 0.79 ^a^	6.17 ± 0.08 ^ac^	18.82 ± 0.75 ^a^	53.29 ± 0.75 ^a^	0.095 ± 0.007 ^ab^
B11	55.39 ± 0.29 ^g^	16.62 ± 0.71 ^ab^	6.76 ± 0.09 ^a^	18.26 ± 0.31 ^ab^	50.66 ± 0.31 ^b^	0.099 ± 0.003 ^a^
B12	55.48 ± 0.22 ^g^	17.22 ± 0.81 ^a^	6.49 ± 0.07 ^ab^	18.41 ± 0.97 ^a^	49.93 ± 0.97 ^bc^	0.086 ± 0.005 ^acd^

Means followed by the same lower letter in the columns (for each kind of starch) do not differ statistically by Tukey test, at 5% of probability.

**Table 3:** Chemical properties of films based on white, red, and black rice starch.

	White Starch (W)		Red Starch (R)		Black Starch (B)	
Code	Water Solubility (%)	WVP ^1^ (g mm/m^2^ dkPa)	Water Solubility (%)	WVP ^1^ (g mm/m^2^ dkPa)	Water Solubility (%)	WVP ^1^ (g mm/m^2^ dkPa)
1	11.10 ± 0.68 ^cd^	1.73 ± 0.36 ^ce^	11.72 ± 0.69 ^de^	1.48 ± 0.21 ^gh^	11.53 ± 0.83 ^f^	8.20 ± 0.12 ^b^
2	6.73 ± 0.91 ^e^	7.85 ± 0.91 ^a^	4.43 ± 0.38 ^g^	2.91 ± 0.56 ^fg^	2.12 ± 0.17 ^i^	15.72 ± 0.48 ^a^
3	9.60 ± 0.27 ^d^	6.33 ± 0.46 ^b^	8.63 ± 0.57 ^f^	1.88 ± 0.47 ^gh^	4.11 ± 0.03 ^gh^	9.32 ± 0.12 ^b^
4	12.04 ± 0.51 ^c^	2.57 ± 0.52 ^c^	12.70 ± 0.32 ^d^	4.06 ± 0.18 ^ef^	10.50 ± 0.24 ^f^	2.83 ± 0.64 ^e^
5	5.89 ± 0.83 ^e^	6.15 ± 0.32 ^b^	3.88 ±0.20 ^g^	12.47 ± 0.47 ^b^	3.34 ± 0.06 ^hi^	3.59 ± 0.35 ^e^
6	9.89 ± 0.40 ^d^	5.20 ± 0.32 ^b^	10.57 ± 0.59 ^e^	6.38 ± 0.38 ^d^	5.05 ± 0.81 ^g^	3.16 ± 0.81 ^e^
7	19.52 ± 0.23 ^a^	0.90 ± 0.01 ^defg^	17.27 ± 0.76 ^b^	5.55 ± 0.23 ^de^	28.14 ± 0.82 ^a^	5.00 ± 0.61 ^d^
8	15.57 ± 0.67 ^b^	2.51 ± 0.46 ^c^	13.00 ± 0.19 ^d^	15.55 ± 0.79 ^a^	18.87 ± 0.77 ^d^	6.59 ± 0.27 ^c^
9	18.54 ± 0.31 ^a^	1.58 ± 0.45 ^cf^	14.89 ± 0.45 ^c^	10.32 ± 0.74 ^c^	21.69 ± 0.19 ^c^	5.58 ± 0.51 ^cd^
10	20.09 ± 0.98 ^a^	0.32 ± 0.01 ^g^	20.66 ± 0.68 ^a^	0.94 ± 0.27 ^h^	23.53 ± 0.27 ^b^	0.60 ± 0.14 ^f^
11	14.66 ± 0.53 ^b^	1.94 ± 0.17 ^cd^	15.85 ± 0.76 ^bc^	12.83 ± 0.90 ^b^	17.15 ± 0.49 ^e^	3.63 ± 0.23 ^e^
12	15.84 ± 0.52 ^b^	0.76 ± 0.16 ^defg^	15.96 ± 0.48 ^bc^	11.71 ± 0.25 ^bc^	20.33 ± 0.30 ^cd^	2.41 ± 0.42 ^e^

^1^ Water vapor permeability. Means followed by the same lower letter, in the columns, do not differ statistically by Tukey test, at 5% of probability.

**Table 4:** Phenolic compounds content (mg EAG/100 g) and antioxidant activity of white, red, and black rice starch films.

Rice Starch	Phenolic Compounds (mg EAG/100 g)	Antioxidant Activity	
		DPPH (g/g DPPH)	ABTS (µM trolox/g)
White	0.58 ± 0.02 ^c^	7.31 ± 0.09 ^a^	5.16 ± 0.10 ^c^
Red	25.26 ± 0.87 ^b^	4.69 ± 0.23 ^b^	14.63 ± 0.14 ^b^
Black	164.96 ± 0.25 ^a^	3.90 ± 0.11 ^c^	20.59 ± 0.09 ^a^

Means followed by the same lower letter (in the columns) do not differ statistically by Tukey test, at 5% of significance.
